# Lipid-Coated Polymeric Nanoparticles for the Photodynamic Therapy of Head and Neck Squamous Cell Carcinomas

**DOI:** 10.3390/pharmaceutics15102412

**Published:** 2023-10-02

**Authors:** Valeri Roschenko, Abdallah M. Ayoub, Konrad Engelhardt, Jens Schäfer, Muhammad Umair Amin, Eduard Preis, Robert Mandic, Udo Bakowsky

**Affiliations:** 1 Department of Pharmaceutics and Biopharmaceutics, University of Marburg, Robert-Koch-Str. 4, 35037 Marburg, Germany; valeri.roschenko@pharmazie.uni-marburg.de (V.R.); abdallah.ayoub@pharmazie.uni-marburg.de (A.M.A.); konrad.engelhardt@pharmazie.uni-marburg.de (K.E.); jens.schaefer@pharmazie.uni-marburg.de (J.S.); muhammad.umairamin@pharmazie.uni-marburg.de (M.U.A.); 2 Department of Otorhinolaryngology, Head and Neck Surgery, University Hospital Marburg, Baldingerstraße, 35033 Marburg, Germany

**Keywords:** human papillomavirus, cancer, PDT, curcumin, HNSCC, PLGA, liposomes, LED

## Abstract

Next to alcohol and tobacco abuse, infection with human papillomaviruses (HPVs) is a major risk factor for developing head and neck squamous cell carcinomas (HNSCCs), leading to 350,000 casualties worldwide each year. Limited therapy options and drug resistance raise the urge for alternative methods such as photodynamic therapy (PDT), a minimally invasive procedure used to treat HNSCC and other cancers. We prepared lipid-coated polymeric nanoparticles encapsulating curcumin as the photosensitizer (CUR-LCNPs). The prepared CUR-LCNPs were in the nanometer range (153.37 ± 1.58 nm) and showed an encapsulation efficiency of 92.69 ± 0.03%. Proper lipid coating was visualized using atomic force microscopy (AFM). The CUR-LCNPs were tested in three HPV^pos^ and three HPV^neg^ HNSCC lines regarding their uptake capabilities and in vitro cell killing capacity, revealing a variable but highly significant tumor cell inhibiting effect in all tested HNSCC cell lines. No significant differences were detected between the HPV^pos^ and HPV^neg^ HNSCC groups (mean IC_50_: (9.34 ± 4.73 µmol/L vs. 6.88 ± 1.03 µmol/L), suggesting CUR-LCNPs/PDT to be a promising therapeutic option for HNSCC patients independent of their HPV status.

## 1. Introduction

Human papillomaviruses (HPVs) are categorized as a group of oncogenic factors for various malignancies, especially cervical and head and neck squamous cell carcinomas (HNSCCs), accounting for approximately 4% of all solid tumors. Additionally, HNSCCs are responsible for 1–2% of all cancer-related mortalities [1,2,3,4]. While the abuse of alcohol and smoking remains the biggest risk factor for HNSCC, approximately 30% of HNSCCs are related to HPV [5]. Yearly mortality rates for HPV-related cancers continue to rise by 2% for men and 1% for women in the United States, highlighting the need for better treatment strategies [6]. Around 80% of HNSCC cases of the oropharynx in men are attributable to HPV-16 [7]. Conventional treatment for HNSCCs involves surgery, radiation, and chemotherapy. However, these therapies may cause adverse effects such as structural deformities, scarring, and irreversible damage to normal tissues. Moreover, chemotherapy may lead to drug resistance, resulting in treatment failure and cancer recurrence [8]. Alternative treatment regimens are being explored, addressing these challenges and aiming to minimize toxicity.

Photodynamic therapy (PDT) holds great promise as an anticancer strategy. PDT is based on activating an otherwise harmless photoactive substance known as a photosensitizer using light of a specific wavelength. When combined with light and oxygen, this photosensitizer generates reactive oxygen species (ROSs), which induce cellular damage, driving cells into apoptosis [9,10,11]. Among various photosensitizers, curcumin is a naturally occurring compound found in the rhizome of *Curcuma longa* L. and has recently gained much attention, as it is well known for its biocompatibility and broad pharmaceutical benefits. Despite its potential as a photosensitizer, curcumin possesses poor water solubility, requiring a suitable drug delivery system (DDS) to enhance its therapeutic efficacy [12,13]. Previous studies have reported that HPV^neg^ HNSCCs tend to increase drug resistance and can be more challenging to treat [14,15]. More so, HPV^pos^ HNSCCs have a better prognosis and treatment outcome after surgery, radiotherapy, and PDT. In particular, patients with HPV^pos^ HNSCCs had a 59% reduction in risk of death from HNSCC after treatment [16,17,18,19].

In this study, we prepared curcumin-loaded lipid-coated polymeric nanoparticles (CUR-LCNPs). CUR-LCNPs consist of curcumin-loaded poly(d,l-lactid-*co*-glycolid) (PLGA) nanoparticles coated with liposomes (LIPOs), formed by 1,2-dipalmitoyl-sn-glycero-3-phosphocholine (DPPC) and 1,2-distearoyl-sn-glycero-3-phosphoethanolamine-N-[methoxy(polyethylene glycol)-2000] (DSPE-PEG_2000_). This hybrid DDS combines the advantages of LIPOs and polymeric nanoparticles, offering increased rigidity, improved uptake, and enhanced biocompatibility, minimizing drawbacks such as instabilities and drug leakage [20]. PLGA is an FDA-approved biodegradable compound widely used as a nanocarrier, while the PEGylated lipid shell can provide longer circulation time inside the body [21,22]. Next to DPPC, which is abundantly present in the mammalian cell membrane and has a phase transition temperature suitable for thermoresponsiveness of liposomes, DSPE-PEG_2000_ can be easily modified to achieve active antibody-targeting properties [23,24,25]. Dynamic light scattering (DLS) and laser Doppler anemometry (LDA) were used for size distribution and surface charge analysis, respectively. Atomic force microscopy was used for morphological characterization, and in vitro cell viability was performed in six different HNSCC lines divided into two subgroups: three HPV^pos^ HNSCC lines and three HPV^neg^ HNSCC lines.

The primary objective of this study was to investigate whether CUR-LCNPs can effectively be deployed for the treatment of HNSCC and if there are differences in the efficacy of this treatment between HPV^pos^ and HPV^neg^ HNSCC cells.

## 2. Materials and Methods

### 2.1. Materials

Curcumin (95%) was purchased from Alfa Aesar (Kandel, Germany). PLGA (Purasorb PDLG 5002A) was obtained from Corbion Purac (Amsterdam, the Netherlands). The 1,2-dipalmitoyl-sn-glycero-3-phosphocholine (DPPC) and 1,2-distearoyl-sn-glycero-3-phosphoethanolamine-N-[methoxy(polyethylene glycol)-2000] (DSPE-PEG_2000_) were ordered from Avanti Polar Lipids (Alabaster, AL, USA). The 2′,7′-dichlorofluorescein diacetate, tetramethylrhodamine ethyl ester (TMRE), and carbonyl cyanide 4-(trifluoromethoxy)phenylhydrazone (FCCP) were purchased from Abcam (Cambridge, UK). The 3-(4,5-dimethylthiazol-2-yl)-2,5-diphenyltetrazolium bromide (MTT), poly(vinyl alcohol) (PVA, Mowiol 4–88), tert-butyl hydroperoxide (TBHP), 2′,7′-dichlorofluorescein diacetate (DCFH-DA), dimethyl sulfoxide (DMSO), and acetonitrile were all supplied by Sigma-Aldrich Chemie GmbH (Taufkirchen, Germany). Acetone was acquired from Fisher Scientific GmbH (Dreieich, Germany). Ultrapure water (PURELAB flex 4, ELGA LabWater, High Wycombe, UK) was used in all experiments.

### 2.2. Light Source

A prototype low-power light-emitting diode (LED) device designed to fit multiwell plates (Lumundus GmbH, Eisenach, Germany) was used for irradiation throughout the experiments. The device was set to a wavelength of 457 nm and 100 mA. Irradiation was performed for 6.5 min, resulting in a radiant exposure of 8.6 J/cm^2^, which was considered optimal based on previous findings on another cancer cell line [26].

### 2.3. Nanoparticle Preparation

Nanoparticles were manufactured using nanoprecipitation [27,28]. Briefly, 2.5 mg of curcumin and 100 mg of PLGA were dissolved in 10 mL of acetone. An amount of 20 mL of 0.5% PVA aqueous solution inside a 50 mL beaker was placed on a magnetic stirring plate (IKA RT 15, IKA-Werke GmbH & Co. KG, Staufen, Germany) set to 600 rpm. The curcumin acetone mixture was injected slowly into the PVA solution. The mixture was immediately protected from light and stirred until the acetone evaporated completely. Afterward, the nanoparticle suspension was adjusted with ultrapure water to 25 mL using a volumetric flask. We prepared 5 mL aliquots, lyophilized them (Christ Alpha 1–4 LSC, Martin Christ Gefriertrocknungsanlagen GmbH, Osterode am Harz, Germany), and stored them at 4 °C. Before each experiment, the lyophilized nanoparticles were redispersed in 5 mL of phosphate-buffered saline (PBS) (pH 7.4) to achieve a theoretical final curcumin concentration of 100 µg/mL. The resuspended nanoparticles were vortexed for 2 min (ZX4 Advanced IR Vortex Mixer, Usmate Velate, Italy) and sonicated for 4 min at room temperature (Elmasonic P, Elma Schmidbauer GmbH, Singen, Germany). Unloaded nanoparticles (Blank NPs) were prepared the same way, the only difference being that they did not contain curcumin.

### 2.4. Liposome (LIPO) Preparation

The LIPOs were prepared using the thin-film hydration method [29]. Briefly, DPPC and DSPE-PEG_2000_ (95:5 mol/mol) were dissolved in a chloroform and methanol 2:1 (*v*/*v*) mixture. The solvents were evaporated at 41 °C with a rotary evaporator (Heidolph Hei-VAP Digital, Heidolph Instruments, Schwabach, Germany) equipped with a vacuum pump. Subsequently, the thin film was rehydrated with 1 mL PBS (pH 7.4) and mixed intensively to form the LIPOs. This liposomal dispersion was then sonicated for 15 min at 45 °C (Elmasonic P, Elma Schmidbauer GmbH, Singen, Germany). Afterward, the liposomal dispersion was extruded 11 times through a polycarbonate membrane with a pore size of 100 nm using an Avanti mini-extruder (Avanti Polar Lipids, Alabaster, AL, USA) at 45 °C to obtain unilamellar LIPOs. The LIPO dispersion was stored at 4 °C until further use.

### 2.5. Preparation of Lipid-Coated Polymeric Nanoparticles (LCNPs)

Previous studies found the optimal lipid-to-polymer ratio to be 1:100 (m/m) [30]. We formed the lipid layer on the surface of the polymeric nanoparticle with a fusion method [31]. Briefly, LIPOs were added to curcumin-loaded polymeric nanoparticles (CUR-NPs) and vortexed for 30 s. Subsequently, the mixture was sonicated for 15 min at 50 °C, resulting in lipid coating on the CUR-NPs’ surface. Unloaded LCNPs were prepared the same way, the only difference being that we used PLGA nanoparticles without curcumin.

### 2.6. Particle Size and ζ-Potential

The hydrodynamic diameter and zeta potential (ζ) of the LIPOs, CUR-NPs, Blank NPs, CUR-LCNPs, and LCNPs were measured with dynamic light scattering (DLS) and laser doppler anemometry (LDA) using Zetasizer Nano ZS (Malvern Panalytica GmbH, Kassel, Germany). Before measurement, the samples were diluted 1:50 (*v*/*v*) with ultrapure water and filled into a folded capillary cell (DTS1070, Malvern Panalytical GmbH) [32]. Three independent samples were analyzed (n = 3).

### 2.7. Encapsulation Efficiency

The encapsulation efficiency (EE) of CUR-LCNPs was determined by comparing the mass of free, non-encapsulated curcumin to the total mass of curcumin [33]. An equal volume of acetonitrile was added to the CUR-LCNPs to dissolve them and determine the total mass of curcumin. The sample was then vortexed and sonicated. Next, the sample was diluted and analyzed spectrophotometrically (Multiskan Go, Thermo Fisher Scientific, Waltham, MA, USA) at λ = 424 nm. The mass of non-encapsulated curcumin was determined after an appropriate volume of CUR-LCNPs was centrifuged at 2000× *g* (Centrifuge 5418, Eppendorf AG, Hamburg, Germany) and washed thrice with PBS (pH 7.4). Afterward, the supernatant was removed carefully, and the remaining curcumin was mixed with acetonitrile (1:1 *v*/*v*) and analyzed as described above. The following Equation (1) was used to calculate the encapsulation efficiency:

(1) $$EE\%=\frac{total\;mass\;of\;curcumin\left[mg\right]-mass\;of\;free\;curcumin\left[mg\right]}{total\;mass\;of\;curcumin\left[mg\right]}\;\%$$

### 2.8. Atomic Force Microscopy

For AFM studies, we analyzed LIPOs, CUR-NPs, and CUR-LCNPs independently. Freshly cut silicon wafers (0.5 cm × 0.5 cm) were mounted onto a glass slide and thoroughly cleaned with isopropanol and ultrapure water. Afterward, LIPOs, CUR-NPs, and CUR-LCNPs were diluted 1:1 (*v*/*v*) with ultrapure water and pipetted onto the silicon wafers. Samples were then incubated for 30 min to allow adherence of the nanovesicles to the surface of the wafer. Excess water was shaken off, and samples were mounted onto the stage of NanoWizard^®^-3 NanoScience AFM (JPK/Bruker, Berlin, Germany), which was vibration-damped and located in an acoustic isolation chamber. The samples were measured at RT in AC mode in air. A target amplitude of 1 V was selected, and the relative setpoint was set to 90%. A commercial AFM cantilever HQ:NSC16/Al BS (MikroMasch Europe, Wetzlar, Germany) with a resonance frequency of 160 kHz and a force constant of 45 N/m was used for all measurements. Settings like gain, setpoint, and drive amplitude were adjusted during measurements to optimize the image quality. Scan speed ranged from 0.6 to 1 Hz depending on image sizes. The raw files were then edited by using JPK data processing Software 4.2. A polynomial fit was subtracted from each scan line independently by using a limited data range. Minor imaging errors were corrected by replacing lines using interpolating, and outliers were replaced using neighboring pixels [30].

### 2.9. Cell Culture 

Six different cancer cell lines were included in this study. All cell lines were derived from head and neck squamous cell carcinoma (HNSCC) and can be divided into two subgroups, i.e., HPV^pos^ and HPV^neg^ cell lines. UM-SCC-47, UPCI-SCC-154, and UM-SCC-104 represent the HPV^pos^ subgroup, while UM-SCC-3, UM-SCC-27, and UT-SCC-26A are the HPV^neg^ subgroup. UM-SCC-47, UM-SCC-104, UM-SCC-3, and UM-SCC-27 were provided by Dr. Carey, University of Michigan, USA. UPCI-SCC-154 was kindly supplied by Dr. Ferris, University of Pennsylvania, USA. UT-SCC-26A cells were furnished by Dr. Grènman, University of Turku, Finland [34,35,36].

Cells were grown under humid conditions at 37 °C and 5% CO_2_ (In-VitroCell ES NU-5841E, NuAire, Inc., Plymouth, MA, USA) and cultured in Dulbecco’s modified Eagle’s medium (DMEM, Capricorn Scientific GmbH, Ebsdorfergrund, Germany) supplemented with 10% fetal bovine serum (Sigma-Aldrich Chemie GmbH) and 1% antibiotic/antimycotic solution (100×, Capricorn Scientific GmbH). Before passaging, using Trypsin (0.05%, Cytiva HyClone™ Trypsin-Protease, Thermo Fisher Scientific) for detachment, the cells were washed with (Ca^2+^/Mg^2+^ free) PBS (pH 7.4, sterile filtered). Subcultures were grown in monolayers until reaching 80% confluence.

The mouse fibroblast cell line (L929), obtained from DSMZ (Braunschweig, Germany), was used to demonstrate biocompatibility. It was cultivated similarly to the cells described above, except Roswell Park Memorial Institute (RPMI) 1640 cell culture medium was used instead of DMEM.

### 2.10. Drug Uptake Studies

#### 2.10.1. Confocal Laser Scanning Microscopy (CLSM)

Confocal laser scanning microscopy (CLSM) was used to analyze the cellular accumulation of curcumin. Briefly, cells were seeded individually in 12-well plates containing cover glasses (ø 15 mm, Paul Marienfeld GmbH & Co. KG, Lauda-Königshofen, Germany) and allowed to adhere overnight. The next day, cells were treated with 10 µmol/L CUR-LCNP for 4 h. After incubation, the cells were washed with PBS and fixed with ROTI^®^ Histofix 4%. Afterward, the cells were washed again and stained with 4′, 6-diamidino-2-phenylindole (DAPI) for 5 min. The cells were washed again, and the cover glasses were mounted on microscope slides and fixed with FluorSave (Calbiochem Corp. San Diego, CA, USA) [28]. Images were captured using a Zeiss Axio Observer Z1 equipped with an LSM 700 confocal unit (Carl Zeiss Microscopy GmbH, Oberkochen, Germany). The samples were excited with λ_ex_ = 405 nm for DAPI and λ_ex_ = 488 nm for curcumin. Appropriate filters were used to obtain the images.

#### 2.10.2. Flow Cytometry

Flow cytometry was executed to evaluate the curcumin uptake of all HNSCC cell lines. Therefore, HNSCC cells were seeded individually in 6-well plates at a density of 3 × 10^5^ cells/well and allowed to adhere overnight. Next, the cells were treated with 10 µmol/L CUR-LCNP for 4 h. Afterward, cells were detached, washed, and resuspended in PBS. For flow cytometry, the Cytoflex LX (Beckmann Coulter GmbH, Krefeld, Germany) was utilized (Filter: B525-FITC; λ_ex_ 488 nm/λ_em_ 525/40 nm). In total, 10,000 single-cell events were recorded in each measurement. FlowJo v10.6.1 was used to further process and analyze the data. Drug uptake values are calculated with the Overton algorithm and presented as percentages of total cells compared to a non-treated control of a similar cell line [37,38].

### 2.11. In Vitro Cell Viability

The different cell types were seeded individually and independently in 96-well plates (Nunclon Delta, Thermo Fisher Scientific, Waltham, MA, USA) at a density of 15,000 cells/0.35 cm^2^ (per well) and were allowed to grow overnight. Afterward, the cells were incubated with the CUR-LCNPs with curcumin concentrations ranging from 50 µmol/L to 0 µmol/L and free curcumin (dissolved in DMSO 1%) for 4 h. After the incubation, the formulations were replaced with fresh medium and immediately irradiated with a light fluence of 8.6 J/cm^2^ at λ = 457 nm before placing them back in the incubator for 18 h. Then, the medium was replaced by MTT reagent diluted with medium 1:10 (*v*/*v*) to a final MTT concentration of 0.2 mg/mL. After incubating the cells for another 4 h, the MTT reagent was replaced with DMSO to dissolve the resulting formazan crystals. The absorbance was measured using the Infinite 200 Pro microplate reader (Tecan Group AG, Männedorf, Switzerland) at λ = 570 nm [39,40].

### 2.12. Biocompatibility Studies

The biocompatibility of CUR-LCNP and CUR-NP was assessed in an MTT assay performed with L929 mouse fibroblast cells. Briefly, L929 cells were seeded in 96-well plates at 15,000 cells/0.35 cm^2^ (per well) and allowed to adhere overnight. Afterward, CUR-LCNP and CUR-NP were added at different concentrations ranging from 0 µmol/L to 50 µmol/L. After the incubation, the formulations were replaced with fresh medium and placed back in the incubator for 18 h. Then, the medium was replaced by MTT reagent diluted with medium 1:10 (*v*/*v*) to a final MTT concentration of 0.2 mg/mL. After incubating the cells for another 4 h, the MTT reagent was replaced with DMSO to dissolve the resulting formazan crystals. The absorbance was measured using the FLUOstar Optima microplate reader (BMG Labtech, Offenburg, Germany) at λ = 570 nm [41].

### 2.13. Mitochondrial Membrane Potential

The mitochondrial membrane potential (MMP) was investigated in UM-SCC-3 cells to determine whether ROS overproduction can be associated with mitochondrial depolarization. Therefore, a TMRE stain was used to detect the PDT-induced reduction in MMP. Briefly, we seeded 15,000 cells/well in 96-well plates and allowed them to grow overnight. Subsequently, we treated the cells with 10 µmol/L of free curcumin (dissolved in 1% DMSO) or an equal concentration of CUR-LCNPs, LCNPs, NPs, and LIPOs for 4 h. The medium was then replaced with fresh medium. Afterward, one plate was irradiated at 8.6 J/cm^2^ while the other was kept in the dark. After 24 h of incubation, the cells were incubated with 400 nmol/L TMRE for 30 min. The positive control was first treated with 50 µmol/L FCCP for 30 min and then stained with TMRE for 30 min like the rest. Next, the cells were washed twice with PBS containing 0.2% bovine serum albumin (BSA), and then fluorescence was measured using a fluorescence spectrophotometer (FLUOstar Optima microplate reader, BMG Labtech, Offenburg, Germany) (λ_ex_ 540 nm/λ_em_ 580 nm) [9].

### 2.14. Cellular Reactive Oxygen Species (cROS) 

DCFH-DA was used to quantify PDT-induced intracellular ROS generation. The cells were seeded in 24-well plates and allowed to grow overnight. The next day, cells were incubated with 10 µmol/L free curcumin (dissolved in 1% DMSO) or equal concentrations of CUR-LCNPs, LCNPs, NPs, and LIPOs for 4 h. The medium was then replaced with 20 µmol/L DCFH-DA in PBS for 45 min under the exclusion of light. Next, the cells were washed twice with PBS, and one plate was irradiated at 8.6 J/cm^2^ while the other plate was kept in the dark. Cells were then lysed and measured using a fluorescence spectrophotometer FLUOstar Optima microplate reader (λ_ex_ 485 nm/λ_em_ 520 nm) [39].

### 2.15. Statistical Analysis

All measurements were conducted in triplicates, and the results are presented as mean ± standard deviation unless otherwise stated. A two-tailed Student’s *t*-test was performed to identify statistically significant differences. Probability values of *p* < 0.05 are considered significant. Statistical differences are denoted as “*” *p* < 0.05, “**” *p* < 0.01, “***” *p* < 0.001, and “****” *p* < 0.0001. GraphPad Prism 9 was used for statistical calculations.

## 3. Results and Discussion

### 3.1. Physicochemical Characterizations

In the present study, CUR-LCNPs were prepared via a fusion method. CUR-NPs were produced using the nanoprecipitation technique. PVA was needed as a stabilizer. LIPOs were prepared via a thin-film hydration method with subsequent extrusion. A PEGylated lipid was incorporated into the formulation to provide additional stability [42,43]. The composition of prepared formulations and their physicochemical properties are displayed in Table 1. CUR-LCNPs reached a high curcumin encapsulation of 92.69 ± 0.03%, which is excellent regarding the drug loading of 2.5% curcumin to PLGA. The hydrodynamic diameters of all formulations are in the nanometric range, and except for LIPOs, all formulations have a narrow size distribution with a PdI < 0.2. In the case of LIPOs, the higher PdI (0.36 ± 0.02) reveals a polydisperse size distribution. All formulations have a negative ζ-potential, with LIPO having the highest negative ζ-potential (−23.03 ± 2.17 mV), and lipid coating slightly decreases the ζ-potential for CUR-LCNPs and LCNPs after fusion. CUR-NPs (149.51 ± 4.35 nm) show a modest increase in size compared to NPs (132.87 ± 0.85 nm), which might be due to the incorporation of the highly hydrophobic curcumin. A tiny increase in hydrodynamic diameter was detected in CUR-LCNPs (153.37 ± 1.58 nm) in comparison with CUR-NPs (149.51 ± 4.35 nm). Given an estimated thickness of the lipid membrane of approximately 4–5 nm, our findings align with the outcomes of prior experiments by our research team [30,44].

### 3.2. Atomic Force Microscopy

**Figure 1:**
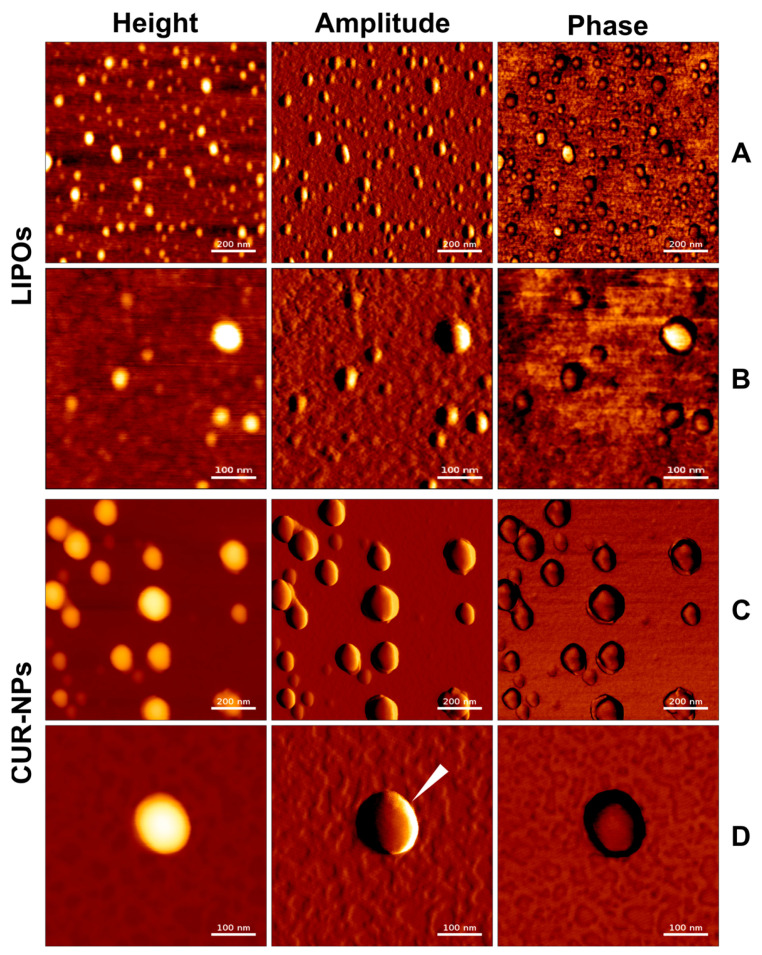
Characterization of nanoparticles by height, amplitude, and phase using atomic force microscopy. (**A**) and (**B**): LIPOs, (**C**) and (**D**): CUR-NPs with an overall smooth and even surface. Scale bars in row (**A**) + (**C**) represent 200 nm. Scale bars in row (**B**) + (**D**) represent 100 nm.

**Figure 2:**
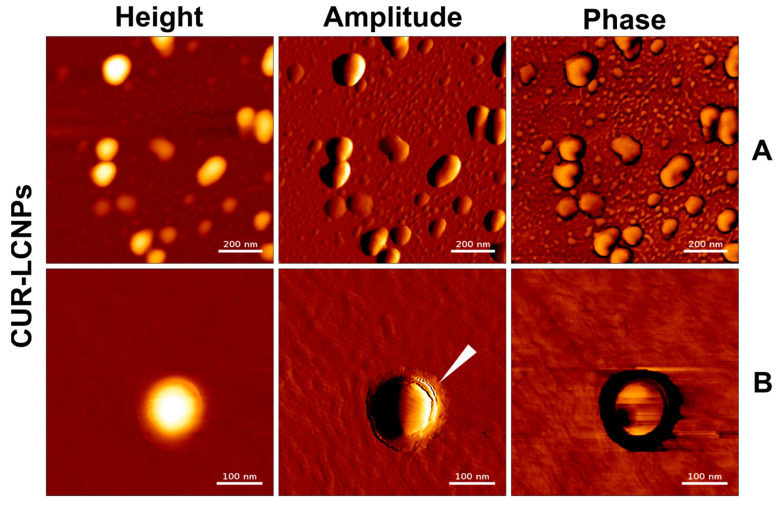
Characterization of nanoparticles by height, amplitude, and phase using atomic force microscopy. (**A**) and (**B**): CUR-LCNPs. The white arrowhead in the amplitude image in **B** indicates a lipid layer on the nanoparticle’s surface. Scale bars in row (**A**) represent 200 nm. Scale bars in row (**B**) represent 100 nm.

The particles were visualized with AFM in AC mode in air. Images were acquired in a height-measured trace, lock-in amplitude, and lock-in phase. Figure 1 and Figure 2 show well-defined spherical shapes for LIPOs, CUR-NPs, and CUR-LCNPs. We present the data as an overview (Figure 1A,C and Figure 2A) and as more detailed images (Figure 1B,D and Figure 2B). Utilizing the height measurement view, we analyzed the size distribution parameters. The size agrees well with the hydrodynamic diameter obtained from DLS studies. In particular, the LIPO spheres were heterogeneous in size, in agreement with the PdI > 0.2 observed with DLS. CUR-NPs and CUR-LCNPs have a homogeneous size distribution of around 150 nm. We visualized the presence of a lipid layer on the surface of the nanoparticles, with some areas around the particle showing incomplete coverage and gaps between the coating, as indicated by the white indicator in Figure 2B. On the other hand, the indicator in Figure 1D shows a particle with a smooth surface, which further supports a successful lipid coating of the polymer particle [30,44].

### 3.3. Drug Uptake Studies

**Figure 3:**
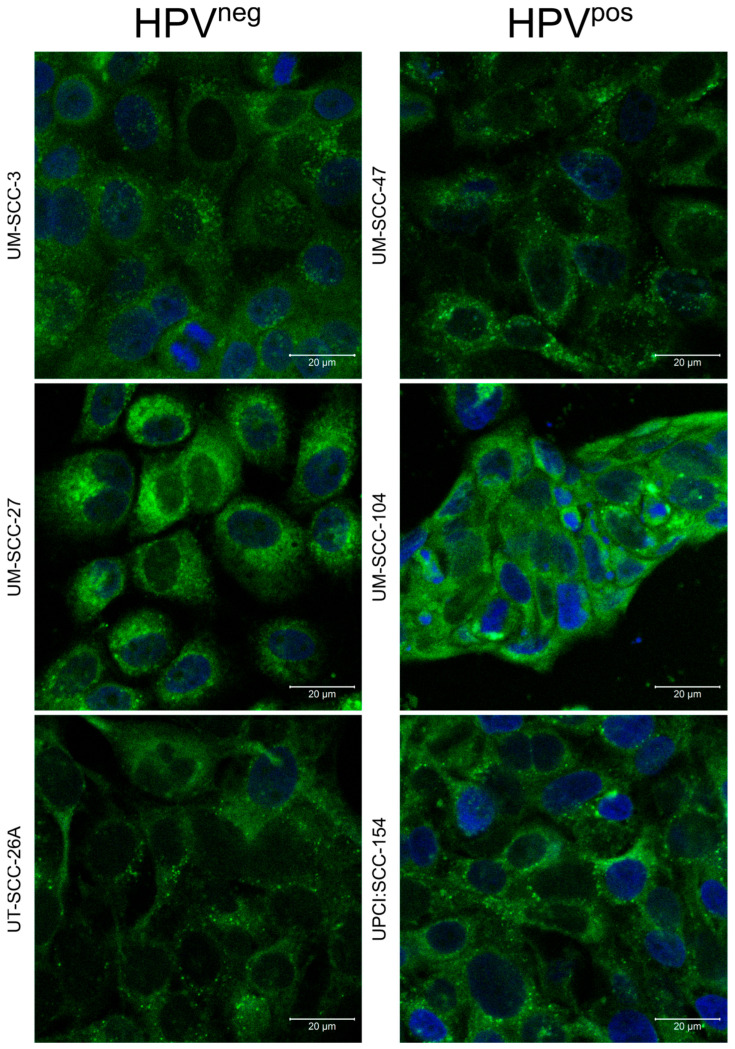
Confocal laser scanning microscope images of six different HNSCC cell lines treated for 4 h with 10 µmol/L curcumin-loaded lipid-coated polymeric nanoparticles (CUR-LCNPs). The merged images show the taken-up curcumin (green fluorescence) and DAPI-stained cell nuclei (blue fluorescence).

**Figure 4:**
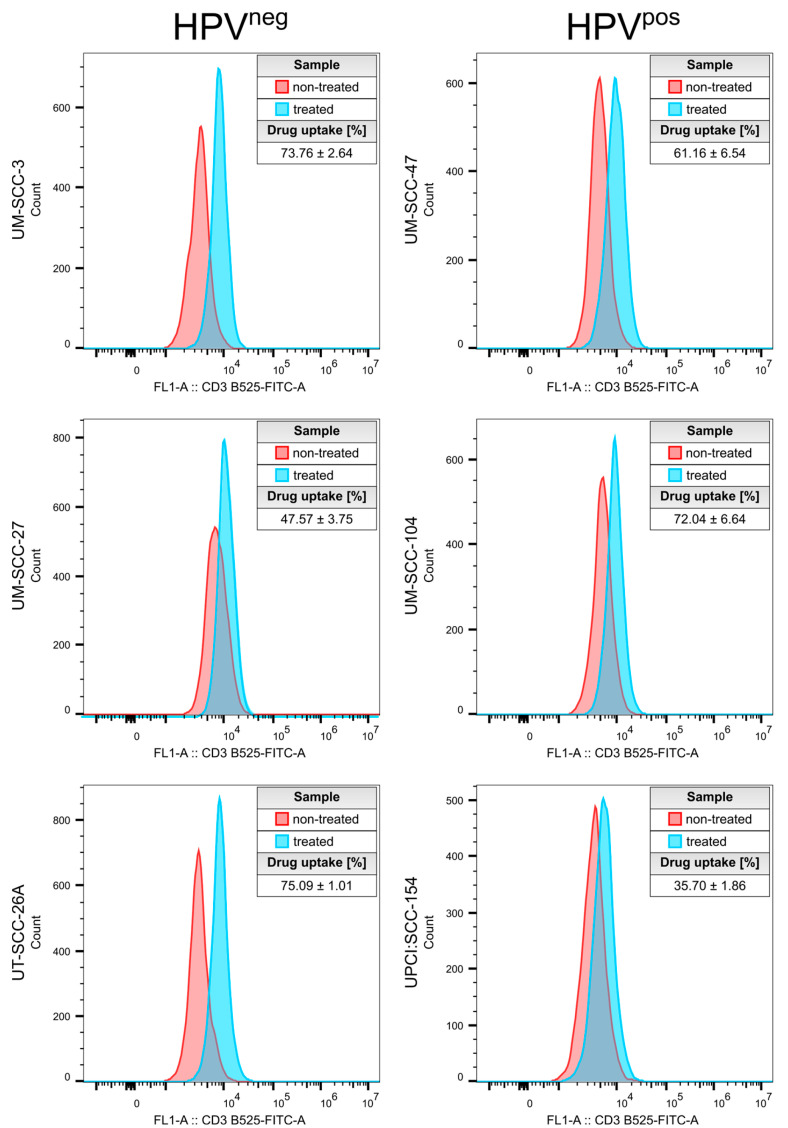
Drug uptake by flow cytometry. Treated (blue) cells were exposed to 10 µmol/L curcumin-loaded lipid-coated polymeric nanoparticles (CUR-LCNPs) for 4 h and compared to non-treated (red) cells. Drug uptake is expressed as mean percentage ± SD, calculated using the FlowJo Overton algorithm.

Drug uptake studies were performed to investigate the capability of CUR-LCNPs to deliver curcumin inside HNSCC cells. Figure 3 shows the merged CLSM images of cells treated with 10 µmol/L CUR-LCNP for 4 h and DAPI staining. All cells show a pronounced curcumin fluorescence (green) in the cytosol of the cells, while DAPI has counterstained the nuclei (blue). Furthermore, flow cytometry was used to quantify the uptake of curcumin inside the HNSCC cells. All six HNSCC cell lines were treated with 10 µmol/L CUR-LCNP for 4 h and subsequently measured, revealing reasonable uptake across all analyzed HNSCC cells (Figure 4). The uptake ranged from 35.70% ± 1.86% in UPCI:SCC-154 cells (HPV^pos^) up to >70% in UM-SCC-3 (HPV^neg^), UT-SCC-26A (HPV^neg^), and UM-SCC-104 (HPV^pos^). There is no significant difference (*p* > 0.05) when the mean of HPV^neg^ (65.47% ± 12.67%) is compared to the mean of HPV^pos^ (53.57% ± 12.69%). The results demonstrate that LCNPs are suitable to deliver highly hydrophobic curcumin to HNSCC cells, independent of their HPV status, and are in line with other studies that reported good uptake of curcumin nanoparticles [38,45]. Based on the enhanced penetration and retention effect, CUR-LCNP could provide suitable accumulation inside solid tumors [46,47].

### 3.4. Effect of Photodynamic Therapy on Cell Viability

**Figure 5:**
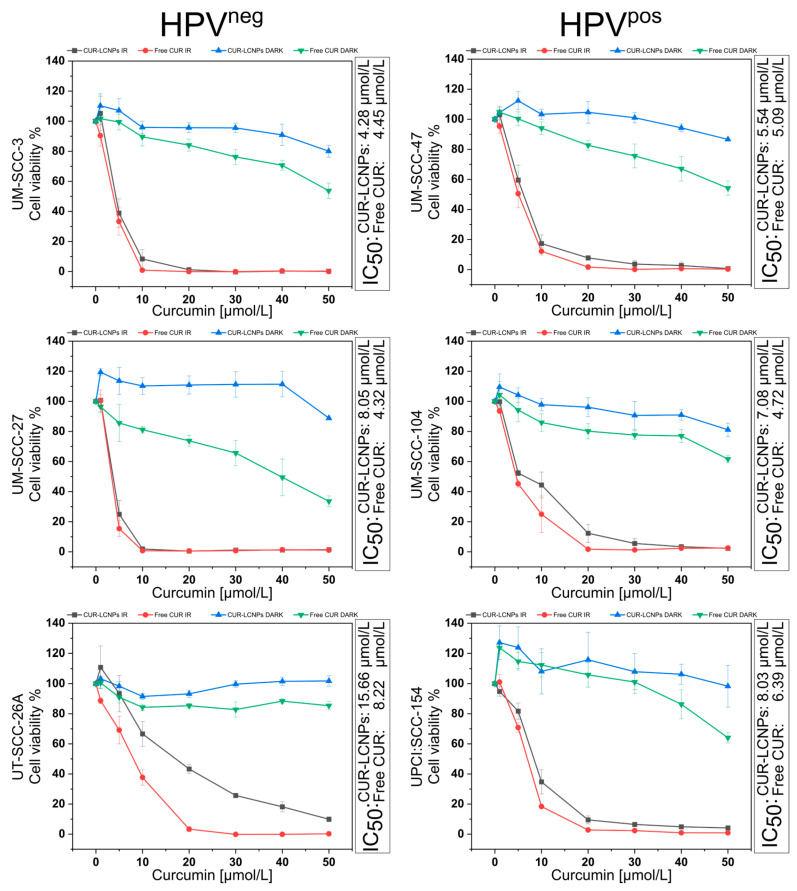
Evaluation of in vitro cell viability via MTT assay in UM-SCC-3, UM-SCC-27, and UT-SCC-26A cells (HPV^neg^ subgroup) and UM-SCC-47, UM-SCC-104, and UPCI:SCC-154 cells (HPV^pos^ subgroup) treated with curcumin-loaded lipid-coated polymeric nanoparticles (CUR-LCNPs) or free curcumin (Free CUR) and irradiated (IR) with λ = 457 nm for 6.5 min, resulting in a radiant exposure of 8.6 J/cm^2^, or without irradiation (DARK). The half maximal inhibitory concentration (IC_50_) was calculated for CUR-LCNP and free CUR (curcumin dissolved in 1% DMSO).

MTT assays were performed to assess in vitro cell viability. Cell viability is calculated by comparing the formazan absorbance of each sample with that of untreated cells. The results of all six cell lines are summarized in Figure 5. Results are split into subgroups of HPV^pos^ and HPV^neg^ cell lines. Furthermore, we compared CUR-LCNPs to free curcumin (dissolved in 1% DMSO) with and without irradiation (dark). The IC_50_ values are calculated via non-linear curve fitting. After 6.5 min of 100 mA irradiation at λ = 457 nm, cells from all tested HNSCC cell lines showed a reduction in cell viability compared to their non-irradiated counterparts. Each cell line shows relatively similar sensitivity to CUR-LCNP at IC_50_ <10 µmol/L, except for CUR-LCNP in UT-SCC-26A. UT-SCC-26A is known to be highly resistant to cisplatin and might be an exciting candidate for further investigations of new therapy strategies [48,49]. Surprisingly, at the same time, UT-SCC-26A showed the greatest curcumin uptake of 75.09% ± 1.01, which leads us to the suggestion that UT-SCC-26A might have a mechanism to successfully withstand different types of therapy that needs to be explored. When we compare the mean IC_50_ (Table 2) of the HPV^pos^ subgroup with the mean IC50 of the HPV^neg^ subgroup, the results show no statistically significant difference (*p* ≥ 0.05), allowing us to conclude that CUR-LCNP/PDT is a promising therapy option for overcoming chemotherapy or irradiation resistance in HPV^neg^ HNSCCs [14,48,50,51,52]. It is worth noting that CUR-LCNP performed very similarly to free curcumin, which we see as in line with the results of our uptake studies. The cells treated with CUR-LCNPs and kept in the dark show only minor dark toxicity when the curcumin concentration is in the highest concentration tested, suggesting that photodynamic activation is key to achieving cell death. On the other hand, cells treated with free curcumin (dissolved in 1% DMSO) show more dark toxicity, which we suggest is due to the increased concentration of DMSO [53]. The results suggest that some cell lines are more sensitive to DMSO than others, with UM-SCC-27 having the greatest impact in terms of DMSO toxicity. Overall, the results show promising data for the potential use of CUR-LCNPs against HPV^pos^ and HPV^neg^ HNSCCs alike. CUR-LCNPs appear effective for PDT treatment of HNSCC tumors while at the same time exhibiting only minor dark toxicity. Additionally, we ranked all HNSCCs regarding their sensitivity to CUR-LCNP/PDT in Table 3. 

### 3.5. Biocompatibility

**Figure 6:**
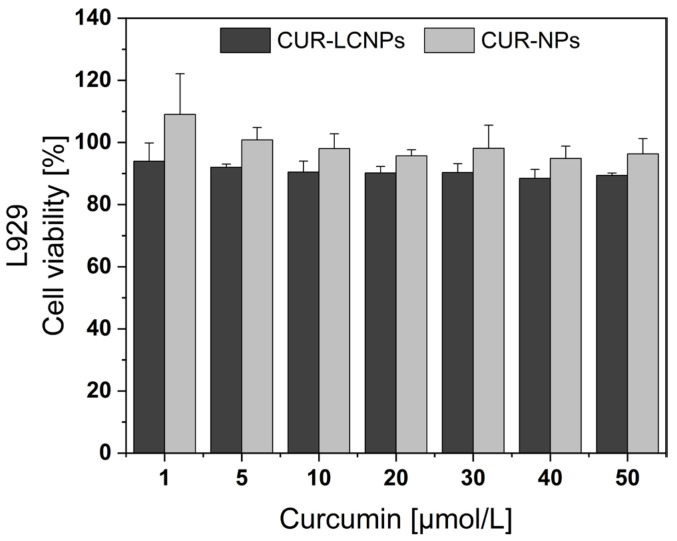
Biocompatibility of curcumin-loaded lipid-coated polymeric nanoparticles (CUR-LCNPs) and curcumin-loaded polymeric nanoparticles (CUR-NPs) with curcumin concentrations ranging from 0 to 50 µmol/L was tested in L929 cells. Cells were kept in the dark throughout the experiment.

MTT assay was performed to assess biocompatibility in L929 mouse fibroblast cells. Cell viability is calculated by comparing the formazan absorbance of each sample with that of untreated cells and is presented in Figure 6. Both CUR-LCNP and CUR-NP showed overall cell viability > 88% and no significant differences (*p* > 0.05) in cell viability. Non-irradiated CUR-LCNP also had very low dark toxicity in HNSCCs (3.3), leading us to the conclusion that CUR-LCNPs are biocompatible [54,55].

### 3.6. cROS

**Figure 7:**
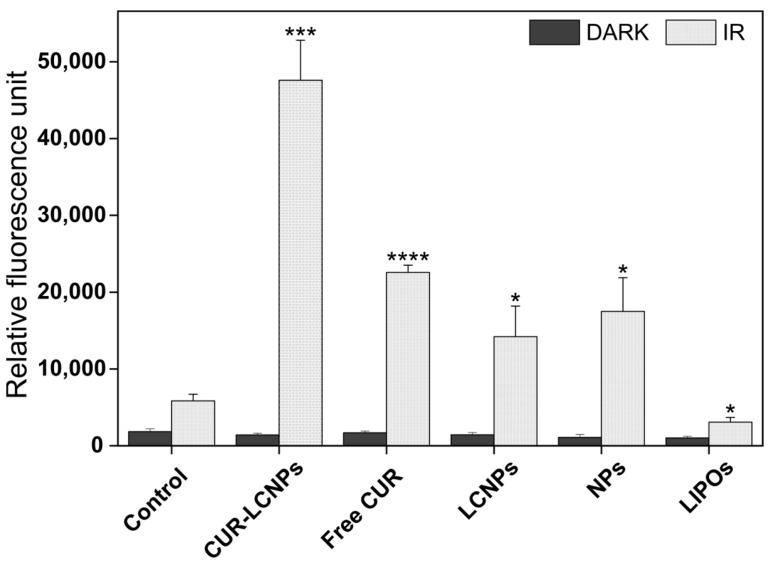
Production of reactive oxygen species inside UM-SCC-3 cells treated with PBS (control), curcumin-loaded lipid-coated polymeric nanoparticles (CUR-LCNPs), free curcumin (free CUR) (curcumin dissolved in 1% DMSO), lipid-coated polymeric nanoparticles (LCNPs), unloaded nanoparticles (NPs), and liposomes (LIPOs) and irradiated (IR) with λ = 457 nm for 6.5 min, resulting in a radiant exposure of 8.6 J/cm^2^, or without irradiation (DARK). Statistical significance is calculated with the relative fluorescence of control IR vs. the respective fluorescence of samples IR. Statistical differences are denoted as “*” *p* < 0.05, “***” *p* < 0.001, and “****” *p* < 0.0001.

To investigate the presence of cROS generation via our photosensitizer, we performed an intracellular ROS generation assay using DCFH-DA. PDT-dependent ROS production is an inducer of apoptosis, which leads to oxidative stress and can lead to cellular apoptosis [56]. This assay was performed in UMSCC-3 cells, a well-established and representative head and neck squamous cell carcinoma cell line with reports dating back to the 1980s [57,58]. In Figure 7, CUR-LCNPs and free curcumin (dissolved in 1% DMSO) induce an extensive increase upon irradiation. In samples without irradiation, there is no noticeable increase of fluorescence and therefore no ROS generation. However, even the samples without curcumin show some increase in fluorescence, even though several previous studies reported those components have no ROS generation [26,59,60]. We suggest the assay might be too sensitive to some extent, and the light source could have an influence on this assay, as it is the only difference compared to non-irradiated samples. 

### 3.7. Mitochondrial Membrane Potential (MMP)

**Figure 8:**
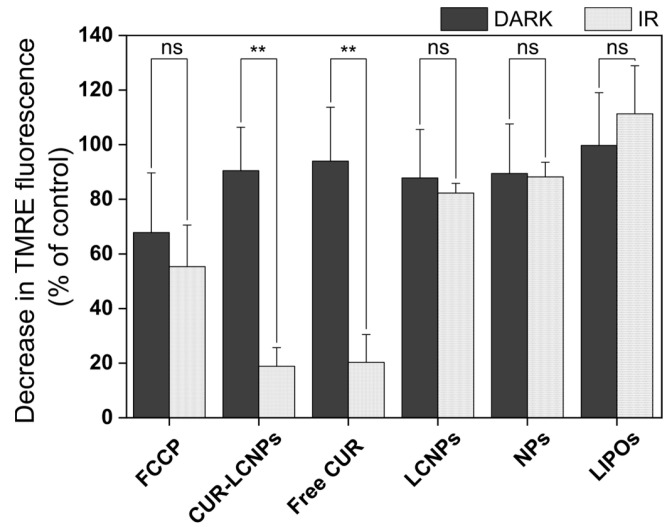
Mitochondrial transmembrane potential of UM-SCC-3 cells treated with FCCP (positive control), curcumin-loaded lipid-coated polymeric nanoparticles (CUR-LCNPs), free curcumin (free CUR) (curcumin dissolved in 1% DMSO), lipid-coated polymeric nanoparticles (LCNPs), unloaded nanoparticles (NPs), and liposomes (LIPOs) and irradiated (IR) with λ = 457 nm for 6.5 min, resulting in a radiant exposure of 8.6 J/cm^2^, or without irradiation (DARK). Results are normalized and compared to PBS as the negative control (100%). Statistical differences are denoted as “**” *p* < 0.01.

An MMP assay was performed to get more insights into the photodynamic effect. TMRE is a cationic cell-permeable dye and accumulates in the negatively charged intact mitochondrial membrane. A fully functional mitochondrial membrane has a potential of up to −200 mV [61]. Changes in MMP can be a sign of mitochondrial damage and also of the cells undergoing apoptosis. These cells have reduced levels of TMRE and therefore reduced fluorescence intensity, which can be quantified considering the control. Our results (Figure 8) show that FCCP (uncoupler of oxidative phosphorylation = positive control) reduced fluorescence to 55.34 ± 15.22% upon irradiation and 67.83 ± 21.85% under dark conditions, while CUR-LCNP reduced fluorescence to 18.85 ± 6.84% and free curcumin (dissolved in 1% DMSO) reduced fluorescence to 20.29 ± 10.20% upon irradiation. There was only a minor reduction under dark conditions of 90.47 ± 15.90% and 93.98 ± 19.74%, respectively, suggesting high disruption of the mitochondria under irradiation. This supports the mechanism of PDT, where curcumin (photosensitizer) and light create cytotoxic ROSs that damage the cell, resulting in apoptosis [56]. Inflicting damage to mitochondria is a well-known target of ROSs generated via PDT [59]. Some researchers even focus solely on mitochondria-targeted PDT [62].

## 4. Conclusions

In conclusion, curcumin-loaded lipid-coated nanoparticles (CUR-LCNPs) were successfully prepared using a fusion method, achieving a high curcumin encapsulation efficiency of 92.69%. Physicochemical characterizations confirmed nanometric size and effective lipid coating. Atomic force microscopy images highlighted the well-defined spherical shape of CUR-LCNPs. Flow cytometry data showed reasonable uptake of curcumin, and the photodynamic efficacy of CUR-LCNPs was evident in inhibiting cell viability across a total of six HNSCC cell lines, both HPV^pos^ and HPV^neg^, with only low dark toxicity. Biocompatibility was shown in L929 cells. Our data indicate that CUR-LCNP-based PDT is a promising minimally invasive and supportive therapeutic approach in head and neck cancer, affecting HPV^pos^ and HPV^neg^ cells equally.

## Figures and Tables

**Table 1:** Summary of particle size (hydrodynamic diameter), ζ-potential, polydispersity index (PdI), and encapsulation efficiency (EE) of CUR-NPs, CUR-LCNPs, NPs, LCNPs, and LIPOs. Results are listed as mean ± standard deviation.

Formulation	Particle Size [nm]	PdI	ζ-Potential [mV]	EE [%]
CUR-NPs	149.51 ± 4.35	0.16 ± 0.06	−2.72 ± 0.31	
CUR-LCNPs	153.37 ± 1.58	0.11 ± 0.01	−3.74 ± 0.31	92.69 ± 0.03
Blank NPs	132.87 ± 0.85	0.06 ± 0.01	−3.10 ± 0.23	
LCNP	134.40 ± 2.78	0.08 ± 0.02	−4.17 ± 0.43	
LIPOs	108.08 ± 4.48	0.36 ± 0.02	−23.03 ± 2.17	

**Table 2:** Summary of half maximal inhibitory (IC_50_) concentration expressed as the mean value of the HPV^pos^ and HPV^neg^ subgroups treated with curcumin-loaded lipid-coated polymeric nanoparticles (CUR-LCNPs) or free curcumin (free CUR). The results are expressed as mean ± standard deviation.

Cell Line	HPV-Status	Mean IC_50_ CUR-LCNPs [µmol/L]	Mean IC_50_ Free CUR [µmol/L]
UM-SCC-3			
UM-SCC-27	HPV^neg^	9.34 ± 4.73	5.66 ± 1.81
UT-SCC-26A			
UM-SCC-47			
UM-SCC-104	HPV^pos^	6.88 ± 1.03	5.40 ± 0.72
UPCI:SCC-154			

**Table 3:** Ranking of all HNSCCs regarding their sensitivity to curcumin-loaded lipid-coated polymeric nanoparticles (CUR-LCNPs)/PDT from highest to lowest.

CUR-LCNP/PDT Sensitivity	Cell Line	HPV-Status	IC_50_ CUR-LCNPs [µmol/L]
Highest	UM-SCC-3	HPV^neg^	4.28
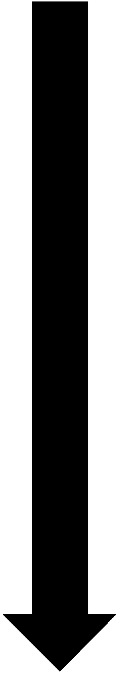	UM-SCC-47	HPV^pos^	5.54
	UM-SCC-104	HPV^pos^	7.08
	UPCI:SCC-154	HPV^pos^	8.03
	UM-SCC-27	HPV^neg^	8.05
Lowest	UT-SCC-26A	HPV^neg^	15.66

## Data Availability

The data presented in this study are available on request from the corresponding author.
